# 

**Published:** 1893-04-22

**Authors:** 


### CONTENTS.

**Should Adults Be Re Vaccinated?** - - - - - - - 49

**Around the Hospitals** - - - - - - - - - 50

**Medical History from the Earliest Times.** By E. T. Withington, M.A., M.B Oxon. LI.—The Reform of Surgery - 51

**Contemporary Theory and Research** - - - - - - 52

**Diet in Disease.** By Mrs. Ernest Hart. XX.—Under-Feeding and Over-Eating - - - - - - - - - - - 53

**Gleanings in Science and Medicine** - - - - - - 54

**Annotations.**—The Leprosy Commission—Herbalism in Yorkshire —Vegetarianism and Consumption - - - - - - - - 55

**Notes and News** - - - - - - - - - - 56

**Festival Dinners.**—King's College Hospital - - - - - 64

**The Hospital Clinic**—Royal Southern Hospital, Liverpool. —The Treatment of Rheumatism - - - - - - - 57

The Hospital for Consumption, Brompton.—The Treatment of Asthma - - - - - - - - - - - - - 59

**The Institutional Workshop**—

Hospital Administration.—The Cost of the Royal Portsmouth Hospital, Portsmouth - - - - - - - - - - 61

Practical Departments. I.—Invalid Chairs - - - - 63

Construction Notes - - - - - - - - - - - 64

**Scraps and Gleanings** - - - - - - - - - - 64

**The Book World of Medicine and Science** - - - - viii

**The Student of Medicine.**—Appointments, Vacancies - x

### EXTRA SUPPLEMENT.—THE NURSING MIRROR.

**News from the Nursing World.**—The Poplar Hospital for Accidents — The Invalid Children's Aid Association — A Popular Home Sister—Trained to Work—Ease in Illness—A Good Report — Hope Deferred — A Bad Example — Manx District Nursing—Kilmarnock—Good Management at Perth —One Nurse Only—The Mysteries of Red Tape—Relief Stations at Chicago—Unwelcome Variety—A Prosperous Irish Hospital—Short Items—The Disabled Nurse - - xxxi

**Sanitation for Nurses.** By a Sanitary Inspector. II.—Notification in Foreign Countries - - - - - - - xxxiii

**Nursing in the Land of Ophir.**—Hospital Experiences in Umtali, Manicaland - - - - - - - - - - xxxiv

**Good Nursing in Dublin** - - - - - - - - xxxiv

**Lifting Patients** - - - - - - - - - - xxxv

**Everybody's Opinion.**—Attainable Perfection—How Not to Nourish Babies—A Correction - - - - - - xxxv

**Death in Our Ranks** - - - - - - - - - - xxxv

**The Jubilee Institute at Taunton** - - - - - - xxxvi

**The East London Hospital for Children** - - - xxxvi

**Training in Children's Hospitals** - - - - - - xxxvi

**Notes and Queries** - - - - - - - - - - xxxvi

**The R.B.N.A. in Edinburgh** - - - - - - - xxxvii

**Nurses in New Jersey, U.S.A.** - - - - - - - xxxvii

**For Reading to the Sick.**—The Prescription. I. - - xxxvii

**Appointments.**—Minor Appointments - - - - - xxxvii

**Her Better Self.**—I. Taking Flight - - - - - - xxxviii

**Wants and Workers** - - - - - - - - - - xxxviii

**The Book World for Women and Nurses** - - - xxxix

